# Altered lipid metabolism in *Drosophila* model of Huntington’s disease

**DOI:** 10.1038/srep31411

**Published:** 2016-08-10

**Authors:** Kumari Aditi, Mallikarjun N. Shakarad, Namita Agrawal

**Affiliations:** 1 Department of Zoology, University of Delhi, Delhi, 110007, India

## Abstract

Huntington’s disease (HD) is late-onset, progressive neurodegenerative disorder caused by expansion of polyglutamine (polyQ) repeat within Huntingtin (Htt) protein. In HD patients, energy-related manifestations such as modulation of weight during entire course of disease with energy deficit at terminal stage have been reported, however, underlying reason remains elusive till date. Lipids, carbohydrate and protein constitute a predominant fraction of body’s energy reservoir and perturbation in their homeostasis may influence weight. To discern role of these energy molecules in weight alteration, we quantified them in an *in vivo* transgenic *Drosophila* model of HD. We document that diseased flies exhibit change in weight due to an altered lipid metabolism, as evident from considerably high lipid levels at the time of disease onset followed by a pathologic decline at end-stage. An alteration in intracellular lipid droplet size suggested altered cellular lipid turnover. Furthermore, diseased flies displayed substantial changes in carbohydrate and protein content. Interestingly, alteration in weight and lipid levels are independent of the feeding pattern in diseased condition and exhibit weak correlation with insulin-like peptide or adipokinetic hormone producing cells. We propose that therapeutic intervention aimed at restoring lipid levels and associated metabolic pathways may improve longevity and quality of patient’s life.

HD is a dominantly inherited, late-onset, progressive neurodegenerative disorder characterized by hallmark chorea, cognitive decline and psychological impairment leading to gradual loss of functional capacity and eventually death. This lethal disease has onset typically in fourth to fifth decade of life with a prevalence of about 1 per 10,000 individuals of European descent^1^,^2^. The causative factor behind HD was assigned to an unstable expansion in the polymorphic trinucleotide (CAG) repeat beyond 35 in Interesting Transcript 15 (IT 15) gene encoding ~348 kD Htt protein, which is ubiquitously expressed^1^. The age of disease onset is inversely related to the number of CAG repeats, with notably enhanced rate of progression at a high copy number^1^,^2^.

The mainstream research in HD focuses on preferential neuronal loss in specific brain areas such as cerebral cortex, caudate and putamen with striatal medium spiny neurons being the most vulnerable, resulting in characteristic clinical symptoms^3^, however, HD pathology encompass wide range of additional metabolic abnormalities including weight loss^4^,^5^,^6^,^7^, energy deficit^8^, progressive alteration in the hypothalamic-pituitary-adrenal axis^9^ and defects in several non-neural tissues such as adipocytes^10^,^11^, muscle^12^,^13^,^14^,^15^, pancreas^16^,^17^ and cardiac cells^15^,^18^. Furthermore, mutant Htt impairs oxidative phosphorylation^19^, glucose homeostasis^20^ and cholesterol biosynthesis^21^,^22^,^23^ leading to an energy deficient background in neuronal and peripheral tissue, as evident in both HD patients and animal models.

Of all the aforementioned manifestations of HD, unintended weight loss despite normal caloric intake^24^,^25^,^26^ remains the most intriguing clinical challenge. One of the principal mechanisms regulating body weight involves a complex interplay of energy intake and energy expenditure^27^, but these factors unlikely explain weight loss in HD. In an attempt to characterize probable reason behind cachexia, several studies have been conducted in R6/2 transgenic mouse model which exhibit many of the cardinal features of HD including weight loss^28^. They provide evidence that R6/2 mouse exhibit altered body weight pattern as a result of progressive alteration in peripheral tissues such as white adipose tissue^10^,^11^ and brown adipose tissue^29^, which play pivotal role in energy reserve accumulation and dissipation, respectively. Adipocytes harbor energy reserves predominantly in the form of lipids which are acquired either directly from diet or synthesized *de novo*. They are known to be key modulators of lipid metabolism and any impairment in fat cells can lead to altered lipid homeostasis. Lipid signaling molecules which in turn efficiently regulate lipid synthesis and breakdown in these cells can potentially be targeted by cellular dysfunction and may further worsen the disease pathogenesis. In an attempt to identify new therapeutic target in HD, an inhibitor of lipid signaling enzyme diacylglycerol kinase ϵ (DGKϵ) was identified which blocked alterations in lipid metabolism and decreased mutant protein accumulation *in vitro*^30^. Moreover, inhibition of DGKϵ in an *in vivo Drosophila* HD model significantly improved the Htt-induced locomotor dysfunction^30^.

The characteristic features of HD can easily be recapitulated in *Drosophila* when exon 1 fragment of mutant Htt with 93 glutamine residues (Httex1p Q93) is expressed in neurons of central and peripheral nervous system^31^,^32^, moreover, key aspects of metabolism remain conserved throughout evolution. In the present study, we observed progressive weight alteration and significant perturbation in the lipid levels of *Drosophila* model of HD when mutant Htt is expressed in all the neurons. We also demonstrate that the impairment in lipid levels is accompanied by a defective cellular lipid accumulation, as evident by drastic change in the size of intracellular lipid droplets present in fat body cells. Additionally, we evaluated the carbohydrate as well as protein moieties in diseased flies and found that their levels varied but does not seem to correlate with the modulation of weight. Nevertheless, to decipher the candidate neurons linked with metabolic abnormalities in HD flies, we investigated the effect of mutant Htt expression in insulin-like peptide producing cells (IPCs) and adipokinetic hormone producing cells (APCs) on body weight, lipid levels and carbohydrate component of flies. Though mutant Htt expression in these neurons leads to considerable modulation in the evaluated parameters, the effect was not as robust and did not correlate substantially with the metabolic changes evident in flies where mutant Htt was expressed in all the neuronal population. Altogether, these findings suggest that progressive weight alteration in HD might be due to an indirect effect of mutant Htt on the integrated process of lipid homeostasis, which may occur due to extensive neuronal impairment mimicking disease condition that becomes more explicit with disease progression. Therefore, therapeutic interventions aimed at lipid metabolic and/or catabolic pathways may not only prove to be effective in delaying the insidious weight changes accompanying disease progression, but the course of disease itself.

## Results

### Flies expressing Httex1p Q93 exhibit alteration in body weight with disease progression

Besides the hallmark neurological symptoms, unstable body mass index (BMI), extensive weight loss despite of normal caloric intake, energy deficit remains a pervasive feature of HD^8^,^33^,^34^,^35^. It is desirable to impersonate this phenomenon in a model organism to address the metabolic alterations associated with insidious weight changes and energy defects. Till date, transgenic *Drosophila* has been used extensively for the study of neurodegenerative diseases as it not only remarkably mimics the hallmark features but is also useful for the identification of effective therapeutics before testing in mice and ultimately in human^36^. In an attempt to study the energy defects associated with HD, we used transgenic flies expressing exon 1 fragment of human Htt protein with expanded polyQ tract (Httex1p Q93) in neurons by using pan neuronal *elav-*GAL4 driver.

**Figure 1:**
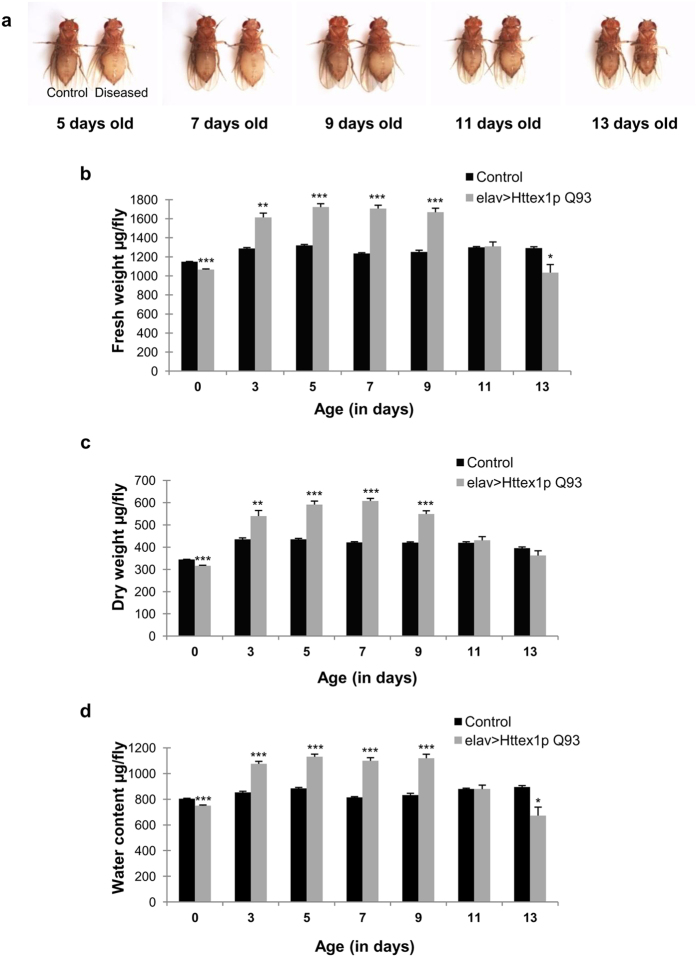
Modulation of body size, weight, dry mass and fluid content in diseased flies. (**a**) The flies on the right are elav>Httex1p Q93 (diseased) and those on the left are control. The diseased flies are heavy at an initial stage (day 5, 7 and 9) and lean at later stage (day 11 and 13). **(b)** Diseased flies (grey bars) show significantly low fresh weight at day 0 followed by an increase in weight from day 3–9 that declined significantly at day 13 as compared to the age-matched controls (black bars). Diseased flies do not survive beyond day 13 post-eclosion. P-value: ***P *<* 0.001; **P *<* 0.01; *P < 0.05. **(c)** Diseased flies (grey bars) display significantly low dry mass at the time of emergence and gain from 3–9 days as compared to the age-matched control flies (black bars). The dry weight is comparable to controls by day 11 and 13. P-value: ***P < 0.001; **P < 0.01; *P < 0.05. **(d)** Diseased flies (grey bars) show significantly low water content upon hatching with increase from day 3–9. The fluid level subsequently declined at day 13 as compared to the age-matched controls (black bars). P-value: ***P < 0.001; **P < 0.01; *P < 0.05.

BMI or body weight serves as a critical indicator of individual’s energy status and any abnormal change in body weight can be the first crucial sign for outlining the difference in energy levels. To better understand the disease and its progression that finally leads to severe weight loss and death, we investigated the energetics of HD flies. We sought to discern the weight profile in normal (UAS-Httex1p Q93 and elav>Httex1p Q20) and diseased flies (elav>Httex1p Q93) as a function of age. A preliminary phenotypic observation and evaluation of body weight was carried out in both the normal and diseased condition. Age-matched normal and diseased flies emerging from a density controlled culture were collected, aged up to 13 days post-eclosion, observed and imaged at different days using Nikon SMZ 1500 microscope. An intriguing pattern of weight alteration was seen in diseased flies with an obese phenotype at an initial stage i.e. at day 5 and at mid-stage i.e. day 7–9; whereas they lost considerable weight and became extremely lean at an advanced stage i.e. day 11–13 (Fig. 1a). Phenotypic variation in the body size was corroborated by subsequent quantification of fresh weight using a microbalance up to thirteen days. Quantification results confirmed that flies expressing Httex1p Q93 became significantly heavier over 3 to 9 days post-emergence (*n* = 50; day 3, *p* = 0.0013; day 5, *p* = 0.00017; day 7, *p* = 8.741E-05; day 9, *p* = 0.0002) followed by significant weight reduction by thirteenth day post-eclosion (*n* = 32; *p* = 0.0378) as compared to age-matched controls. Control flies became significantly heavier by 3 days of emergence compared to their day 0 counter parts (*n* = 50; *p* = 5.06E-05) (Fig. 1b). These results showed that diseased flies exhibit a characteristic pattern of weight change throughout disease progression with marked resemblance to HD patients. These predominant changes in body weight could be a reflection of the energy levels in HD pathogenesis.

In an attempt to further investigate the alterations in energy reserves with disease progression, we evaluated the dry weight as well as water content in HD flies from disease onset to progression. Dry weight is indicative of lipids, glycogen, protein and other structural components and constitutes a major fraction of body weight. In diseased flies, dry weight was significantly high from day 3 to 9 as compared to age-matched controls (*n* = 50; day 3, *p* = 0.01173; day 5, *p* = 0.00034; day 7, *p* = 3.0301E-05; day 9, *p* = 0.00058), but became comparable to normal on day 11 and 13. Similarly, diseased flies had significantly high water content from day 3 through day 9 compared to controls (*n* = 50; day 3, *p* = 7.1313E-05; day 5, *p* = 8.9086E-05; day 7, *p* = 0.00016; day 9, *p* = 0.00019) that significantly reduced by day 13 (*n* = 32; *p* = 0.02713) (Fig. 1c,d). These results further strengthen that the levels of major macromolecules remain altered in HD flies and might be one of the contributing factors in HD progression.

### Diseased flies display dysregulated food intake

**Figure 2:**
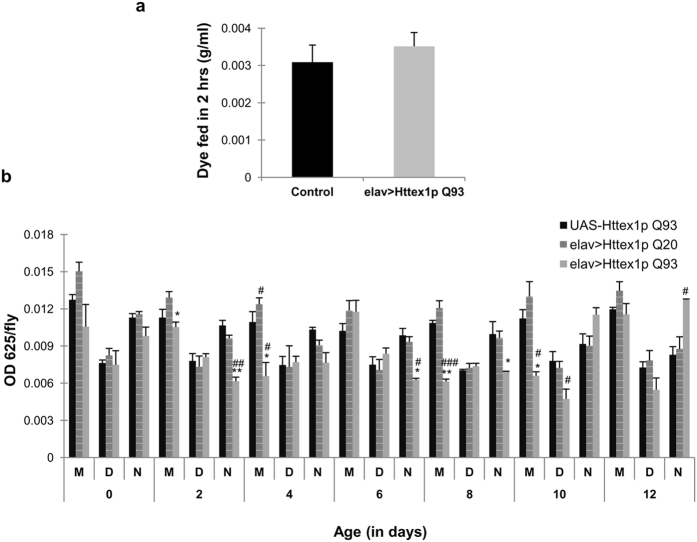
Arrhythmic feeding behavior of HD flies. (**a**) Normal (black bars) and diseased (grey bars) third instar larvae show comparable food intake. **(b)** Diseased flies (grey bars) show differential food intake as compared to age-matched controls (black bars and pattern bars) monitored from day 0–12. Black bars represent non-expressing UAS-Httex1p Q93 flies and pattern bars represent unexpanded elav>Httex1p Q20 flies that serves as control. M, D & N denotes morning, day and night, respectively. **#** represents comparison between diseased and control flies; *represents comparison between elav>Httex1p Q93 (diseased) and unexpanded flies. P-value: ***P < 0.001; **P < 0.01; *P < 0.05.

We monitored food intake of healthy, unexpanded elav>Httex1p Q20 and diseased elav>Httex1p Q93 larvae and flies to understand if differential weight gain and loss as compared to healthy flies can be attributed to their feeding behavior. Diseased larvae had comparable feeding pattern to that of the control (*n* = 30) (Fig. 2a). However, a systematic evaluation of food intake during adult stage at different time points i.e. morning, day and night at an interval of 8 hours under *ad-libitum* access to food revealed that diseased flies show arrhythmic pattern of food intake as compared to age-matched controls. We found that during daytime, at an early age, diseased flies fed comparable to controls that significantly declined at later stages (*n* = 15; day 10, *p* = 0.0468). However, during morning and night time, diseased flies exhibited differential pattern of feeding throughout the disease as compared to the age-matched control flies (Fig. 2b). Contrary to our assumption, the feeding pattern of diseased flies does not seem to contribute to their obese phenotype observed at an initial stage of disease followed by significant weight loss as disease reaches to its terminal stage.

We also observed accumulation of an excess of energy reserves at initial stage of disease followed by depletion at the later ages regardless of their feeding behavior suggesting that they do not seem to alter their feeding pattern in response to fluctuating energy reserves with disease progression.

### Alteration of carbohydrate and protein level in diseased condition

We observed a unique pattern of weight change in diseased flies associated with disease onset and progression. We further extended our observation to decipher the relationship between weight modulation and energy accumulation through metabolic alteration in diseased condition. Apparently, an increase or decrease in weight beyond the normal limits can be mediated through conspicuous changes in levels of several molecular moieties such as lipids, glycogen, protein and water content^37^,^38^,^39^. Therefore we evaluated glycogen, protein and other structural moieties to affirm their status in diseased condition.

**Figure 3:**
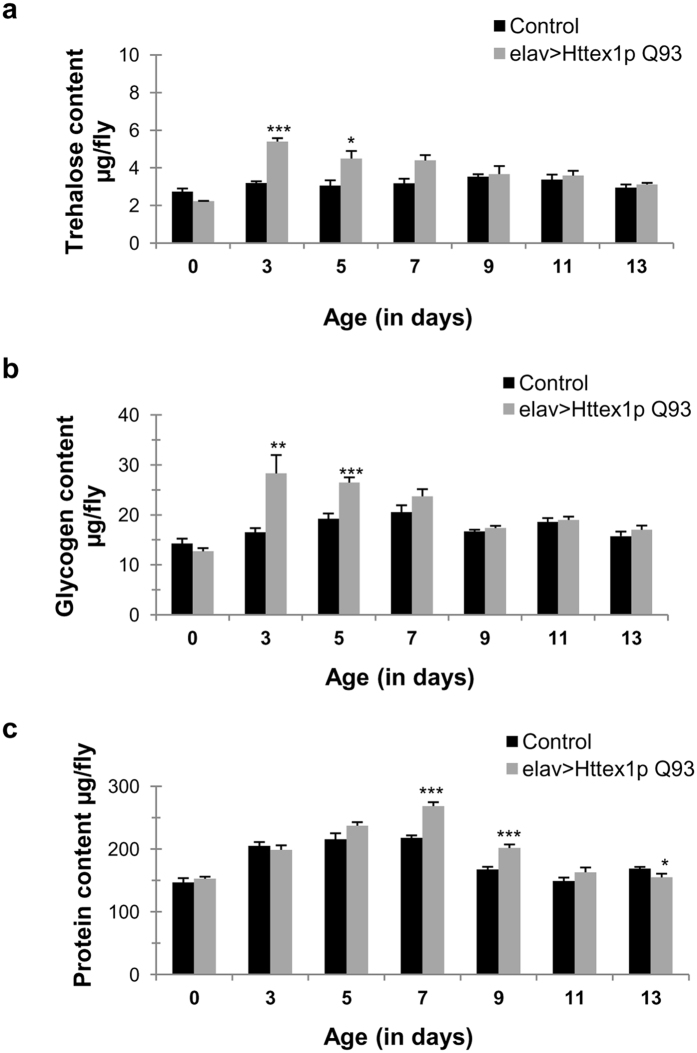
Altered trehalose, glycogen and protein levels in HD flies. (**a**) elav>Httex1p Q93 flies (diseased, grey bars) have significantly high circulating hemolymph sugar at an initial stage of disease (day 3–5) that becomes comparable to age-matched controls (black bars) at later stages (day 7–13). P-value: ***P < 0.001; **P < 0.01; *P < 0.05. **(b)** Diseased flies (grey bars) exhibit significantly high glycogen levels at 3–5 days of age, however, at later stage it is comparable to age-matched controls. P-value: ***P < 0.001; **P < 0.01; *P < 0.05. **(c)** Diseased flies (grey bars) display significantly high protein content upon disease progression (day 7–9) with significant decline at day 13 as compared to age-matched control flies (black bars). P-value: ***P < 0.001; **P < 0.01; *P < 0.05.

To obtain a global picture of metabolic changes induced by neuronal expression of mutant Htt protein, we examined the levels of major energy reserves such as glycogen, circulating sugar and protein. We found that diseased flies exhibit additional metabolic changes along with dysregulated body weight. Evaluation of glycogen, protein and hemolymph sugar mainly in the form of trehalose depicts significant changes through disease progression. Trehalose content varied in accordance with the glycogen levels in diseased flies. We presume that in diseased condition, flies accumulated trehalose and glycogen reserves during the initial phase of disease, with maximal levels at day 3 (glycogen *n* = 20, *p* = 0.004291; trehalose *n* = 20, *p* = 4.7586E-08) and day 5 (glycogen *n* = 20, *p* = 1.0466E-05; trehalose *n* = 20, *p* = 0.01270) (Fig. 3a,b) but thereafter, these energy molecules are comparable to their age-matched controls until day 13. Control flies displayed a significant increase in glycogen content during the first week of adult life (*n* = 20; *p* < 0.01), as mentioned in previous studies^40^. Conversely, we found that the protein profile of diseased flies matched with the controls during the initial phase of disease, however, it reaches a peak at day 7 (*n* = 20; *p* = 7.32385E-07) and declines significantly afterwards (*n* = 20; day 13, *p* = 0.0490) (Fig. 3c). Our results show that the control flies had increased protein content during the first week, as most of the adult tissues such as fat body, gut and ovary are fully developed during this phase. Further, significant increase in protein content in diseased flies could be due to their bigger size at day 7 and 9 as compared to their controls (Fig. 1a).

### Weight change is modulated by total lipid levels in HD flies

Lipid synthesis, storage and breakdown predominantly influences the process of weight change in organisms and maintenance of lipid homeostasis involves monitoring and control from various organs such as brain, liver and adipose tissue. Coordination by brain employs several regulatory centers that are able to sense the increase or decrease in fat reserves in accordance to body’s energy needs and respond to it by regulating the feeding and metabolic processes. The basic regulatory components remain conserved between *Drosophila* and human^41^,^42^,^43^. Any alteration in these regulatory centers by mutant Htt protein may lead to altered weight regulation and defective metabolism, as evident in our experimental model. Therefore, to precisely illuminate the role of altered metabolism in weight regulation, we focused on characterization of lipid levels in the disease context. We evaluated lipid-free dry weight and global lipid levels in HD flies from disease onset to progression.

**Figure 4:**
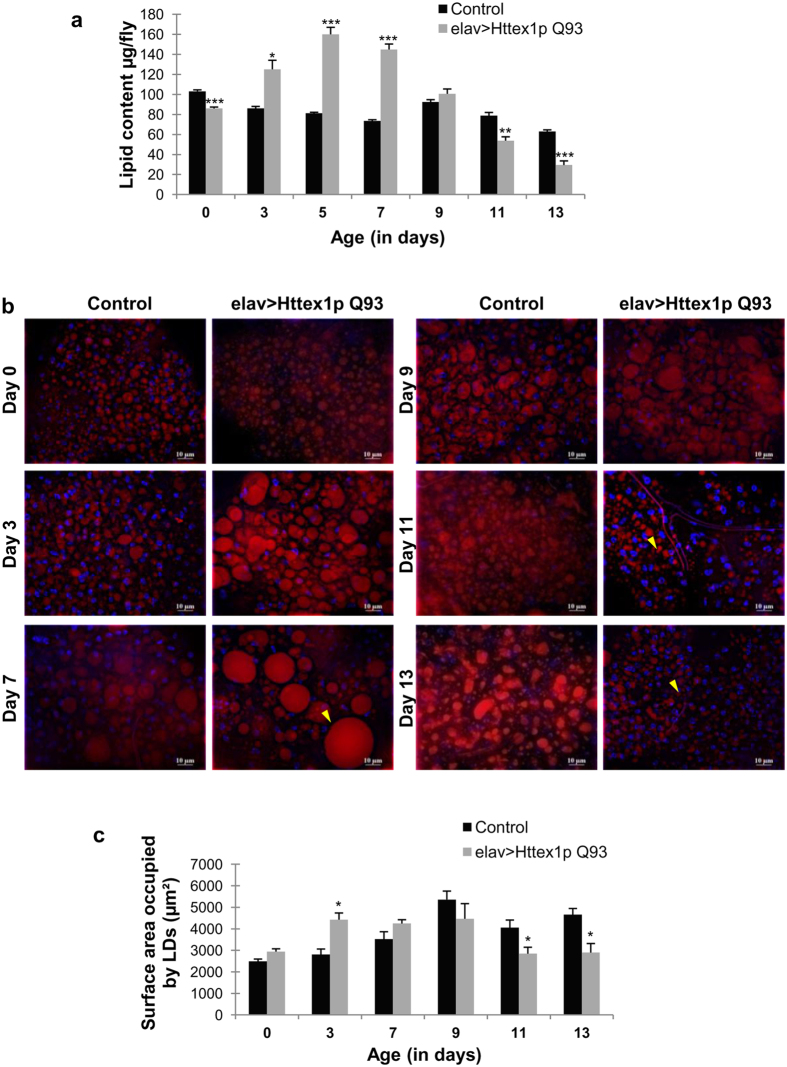
Dysregulated lipid levels and lipid droplets in HD flies. (**a**) elav>Httex1p Q93 flies (diseased, grey bars) have significantly low lipid content at the time of emergence that shoots up upon disease progression (day 3–7). The lipid level becomes significantly low at terminal stage of the disease (day 11–13) as compared to the age-matched control flies (black bars). P-value: ***P < 0.001; **P < 0.01; *P < 0.05. **(b)** Lipid droplets (LD) in control flies did not undergo much variation in their size whereas diseased flies had bigger lipid droplets in abdominal fat cells at day 7. At the later stage of disease (11–13 days) the size and intensity of LD is significantly reduced as compared to their age-matched controls. Arrowheads mark large LD at day 7 and smaller LD at day 11–13. Scale bar: 10 μm. **(c)** Quantification of total surface area of LD representing significant increase in area occupied by lipid droplets at day 3 followed by decline at day 11 and 13 in diseased condition (grey bars). Error bars represent ± SEM. P-value: *P < 0.05.

Lipid-free dry mass represents glycogen, protein and the remaining structural components. These components were significantly high in diseased flies from day 3 to 9 (*n* = 50; day 3, *p* = 0.01198; day 5, *p* = 0.00093; day 7, *p* = 9.3191E-05; day 9, *p* = 0.00029) as compared to controls, but became comparable to age-matched controls on day 11 and 13. Furthermore, global lipid levels of diseased flies were significantly higher at the time of disease onset i.e. by 3 to 7 days (*n* = 50; day 3, *p* = 0.01140; day 5, *p* = 0.00030; day 7, *p* = 0.00011) compared to age-matched controls, whereas it became significantly low on day 11 (*n* = 50; *p* = 0.00121) and day 13 (*n* = 32; *p* = 0.00055) during which disease progression presumably accelerates (Fig. 4a). Control flies display a rhythmic decline of lipid content over seven days (*n* = 50; *p* < 0.01). These results clearly suggest that lipid levels, in addition to water content and other vital components undergo extensive alteration throughout disease progression. Pathologic increase followed by steep decline in lipid levels may severely impact the overall health of challenged individuals by implicating vital processes such as inter and intracellular signaling, thermo genesis and/or oxidative stress. Above results imply that metabolic processes pivotally linked with lipid homeostasis remain altered in the diseased condition, leading to discrepancy in body weight.

### Dysregulated intracellular lipid accumulation in HD flies

We found that *Drosophila* model of HD exhibited altered lipid levels with disease progression. Even though we expressed the mutant protein only in neurons, peripheral lipid metabolism appears modulated as evident by high systemic lipid levels followed by low lipid levels at the terminal stage. To test whether pan-neuronal expression of mutant Htt causing neurodegeneration influences lipid accumulation in peripheral tissues, we measured the distribution of lipid droplets in abdominal fat body from disease onset to progression. We found that expression of mutant Htt exclusively in neurons also influenced lipid droplet distribution in peripheral fat tissue such as abdominal fat body, in agreement with whole-body lipid level. We observed that the lipid droplet size remains unaltered in larval fat body (*n* = 6). Interestingly, we found considerably large lipid droplets in abdominal fat body of 3–7 days old adults followed by substantially small lipid droplets by day 11–13 (*n* = 6) (Fig. 4b,c). We found an alteration in the size of lipid droplets during the course of the disease without targeting expression of mutant Htt in abdominal fat body implying that dysregulated lipid metabolism is the consequence of disease condition. Taken together, our results suggest that mutant Htt expression in neurons lead to neurodegeneration along with the retardation of peripheral lipid metabolism.

### Effect on metabolic activity by expression of mutant Htt in IPCs

Expression of mutant Htt in all the CNS and PNS neurons demonstrated abnormal changes in body weight, major energy reservoirs as well as intracellular lipid depots in challenged flies. Intriguingly, to answer the key question of how neuronal Httex1p Q93 expression results in these metabolic abnormalities, it is crucial to investigate the role of candidate neurons which may be associated with Httex1p Q93 induced metabolic defects. To elucidate the mechanistic basis of metabolic abnormalities evident in diseased flies, we extended our work by targeting mutant Htt expression in two discrete neuronal groups such as IPCs and APCs, using *Drosophila* insulin-like peptide 2 (Ilp2) and adipokinetic hormone (Akh) specific drivers, respectively.

**Figure 5:**
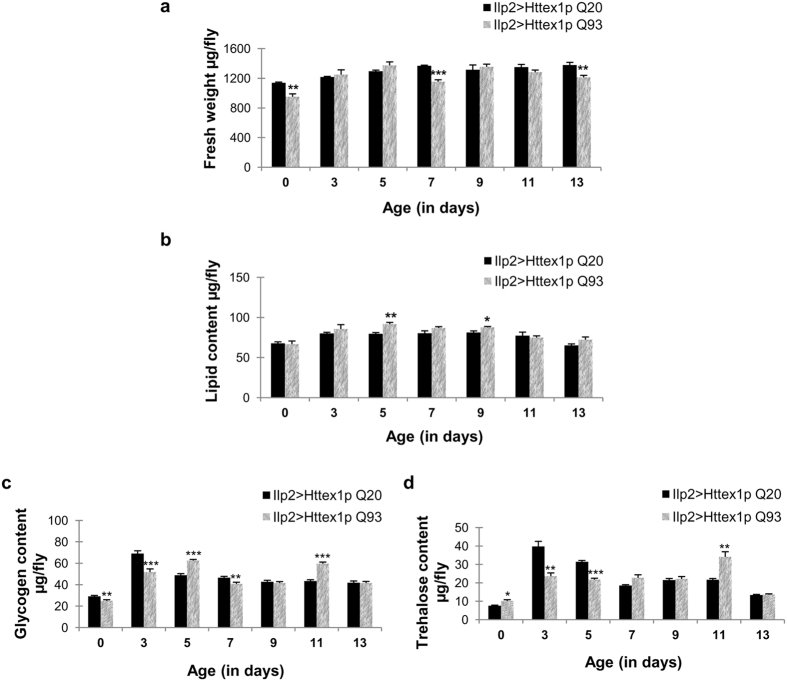
Modulation of fresh weight and metabolic activity by mutant Htt expression in insulin-like peptide 2 producing cells. **(a)** Flies expressing Httex1p Q93 exclusively in Ilp2 producing cells (pattern bars) show significantly lower fresh weight at day 0, 7 and 13 as compared to the age-matched control flies (black bars). P-value: ***P < 0.001; **P < 0.01; *P < 0.05. **(b)** Significantly high lipid content at day 5 and 9 as compared to age-matched controls. P-value: ***P < 0.001; **P < 0.01; *P < 0.05. **(c)** Significantly low glycogen content at 0, 3 and 7 days which becomes significantly high at day 5 and 11. P-value: ***P < 0.001; **P < 0.01; *P < 0.05. **(d)** High circulating trehalose levels at day 0 and 11 and significantly low levels at day 3 and 5. P-value: ***P < 0.001; **P < 0.01; *P < 0.05.

IPCs are one such group of neuroendocrine cells which profoundly modulate sugar and lipid metabolism along with several other biological processes such as growth, aging and metabolic homeostasis^41^,^44^. These cells predominantly produce Ilp 2 along with three other neuropeptides Ilp 1, 3 and 5^41^. We targeted Httex1p Q93 expression in *Drosophila* IPCs from embryonic stage onwards and evaluated their fresh weight, lipid content as well as carbohydrate fractions for up to thirteen days. Flies expressing Httex1p Q93 in IPCs displayed significantly low fresh weight at day 0 (*n* = 25; *p* = 0.00665), 7 (*n* = 45; *p* = 0.00056) and 13 (*n* = 50; *p* = 0.00652), however, the lipid content of these flies was comparable to age-matched controls from 0 to 13 days, with significant increase only at day 5 (*n* = 41; *p* = 0.00257) and 9 (*n* = 28; *p* = 0.0279) (Fig. 5a,b). In addition, carbohydrate estimation revealed significantly low glycogen levels at day 0 (*n* = 12; *p* = 0.0055), 3 (*n* = 12; *p* = 0.00011) and 7 (*n* = 12; *p* = 0.00547), with significant increase at day 5 (*n* = 12; *p* = 7.1922E-08) and 11 (*n* = 12; *p* = 5.86719E-09) (Fig. 5c). Moreover, to clearly depict the impact of Httex1p Q93 expression in IPCs on circulating sugar levels, we quantified trehalose levels and found significantly high trehalose content at day 0 (*n* = 12; *p* = 0.01022) and 11 (*n* = 12; *p* = 0.005806) with significant decline at day 3 (*n* = 12; *p* = 0.00101) and 5 (*n* = 12; *p* = 2.55142E-06) as compared to control flies. From day 7–9 and day 13, trehalose levels of diseased flies remained comparable to control flies (Fig. 5d).

From these results, we speculate that flies expressing mutant Htt exclusively in IPCs display changes in metabolic activity at very few time points that are different than the mutant Htt expression in all the neuronal population. Expression of mutant Htt in IPCs are mostly capable of maintaining a steady state homeostasis in their body weight, lipid and trehalose content except modulation in these parameters at specific ages. Besides, these flies did not show any defect in their growth or developmental time upon mutant Htt expression which is further indicative of functional insulin supply from IPCs^41^.

### Httex1p Q93 expression in APCs modulates body weight, lipid and carbohydrate moieties

**Figure 6:**
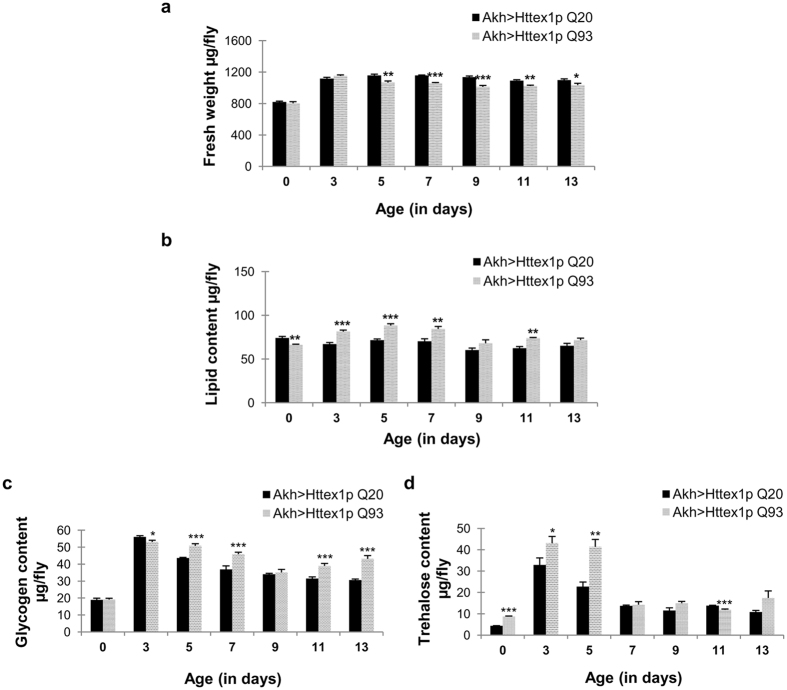
Altered body weight, lipid content and carbohydrate in flies expressing mutant Htt in Akh specific neurons. (**a**) Flies expressing Httex1p Q93 under the control of Akh driver (pattern bars) have significantly low fresh weight from day 5 to 13 as compared to age-matched control flies (black bars). P-value: ***P < 0.001; **P < 0.01; *P < 0.05. **(b)** Significantly low lipid content at day 0, followed by increase in lipid content at day 3, 5, 7 and 11. P-value: ***P *<* 0.001; **P *<* 0.01; *P < 0.05. **(c)** Significantly low glycogen content at day 3 which increases at day 5, 7, 11 and 13 in comparison to age-matched controls. P-value: ***P *<* 0.001; **P *<* 0.01; *P < 0.05. **(d)** Significantly high trehalose levels at day 0, 3 and 5 that declines at day 11. P-value: ***P *<* 0.001; **P *<* 0.01; *P < 0.05.

Using the same experimental regimen mentioned previously, mutant Htt was expressed under the control of Akh driver to investigate role of these neuroendocrine cells in the regulation of lipid and carbohydrate metabolism in HD. APCs produce glucagon like neuropeptides which facilitate the mobilization of lipids and glycogen stores^45^. Fresh weight quantification of these flies revealed their significantly low body weight from 5 to 13 days (*n* = 50, day 5, *p* = 0.00844; *n* = 50, day 7, *p* = 2.0135E-05; *n* = 45, day 9, *p* = 0.000644; *n* = 50, day 11, *p* = 0.00154; *n* = 48, day 13, *p* = 0.04710) as compared to age-matched control flies (Fig. 6a). However, the lipid content of these flies was significantly low at the time of emergence (*n* = 39; *p* = 0.0045) and high from day 3 to 7 (*n* = 50; day 3, *p* = 0.00045; day 5, *p* = 0.00026; day 7, *p* = 0.00781) and also at day 11 (*n* = 50; *p* = 0.00198) (Fig. 6b). Furthermore, carbohydrate estimation depicted significantly low glycogen levels at day 3 (*n* = 12; *p* = 0.0217) which increased significantly from day 5 to 7 (*n* = 12; day 5, *p* = 3.9E-05; day 7, *p* = 0.00101) and then at 11 to 13 days of age (*n* = 12; day 11, *p* = 0.00029; day 13, *p* = 7.3E-06) (Fig. 6c). Nonetheless, circulating sugar trehalose levels were also significantly high up to 5 days after emergence (*n* = 12; day 0, *p* = 4.80E-07; day 3, *p* = 0.0487; day 5, *p* = 0.00144) that becomes comparable to normal level at day 7, 9 and 13, with significant decline at day 11 (*n* = 12; *p* = 0.00078) (Fig. 6d). Altogether, these results suggest that flies expressing Httex1p Q93 specifically in APCs undergo considerable alteration in their body weight, however, interestingly major energy reserves in the form of lipids and carbohydrate fractions seem to be extensively modulated at specific ages.

## Discussion

Most predominant neuropathological changes in HD involve prominent cell loss and atrophy of specific sets of neurons in caudate, putamen and cerebral cortex. These neurons are more susceptible to damage and give rise to the characteristic clinical symptoms. In addition to the neurobiological abnormalities, metabolic impairments such as weight loss with altered energetics remained important clinical challenge and suspected to enhance disease progression in patients. Body weight alteration with HD progression has previously been recognized in human patients suggesting that at an early stage of the disease, HD subjects have lower than normal body mass index^5^, and they continue to lose weight despite high caloric intake^4^. Studies in HD mouse model also demonstrate a typical pattern of weight alteration, where they undergo severe wasting at ~12 weeks of age despite having enhanced adiposity at an initial stage (8–9 weeks)^10^,^11^. In the present study, we demonstrate a characteristic weight change phenomenon in an *in vivo* transgenic *Drosophila* HD model. A phenotypic and quantitative inspection of body weight ascertained that diseased flies undergo severe alterations in weight with HD progression as evident by lower body weight at the time of emergence, rapid weight gain as disease symptoms become evident followed by extensive weight loss at the later or terminal stage. Drastic weight alteration may occur as a result of compromised physical activity, altered fat deposition, abnormal glycogen accumulation, fluid imbalance, caloric intake differing from body’s energy demand, metabolic abnormality or a combination of all these factors. From the feeding analysis of diseased flies, we speculate that abnormal weight pattern in diseased flies occur independent of their feeding behavior, and a hyper or hypo-phagic condition is not responsible for excessive weight gain or loss.

In an attempt to find the metabolic components inflicted in weight change in human patients, several markers have been identified till date, but the mechanism contributing to weight change remains unclear and possibly multifaceted. In addition, *in vivo* confirmation remains indispensable as it offers a complex system with advanced level of metabolic integrity that can unravel novel aspects of disease pathogenesis. Here, we carried out a comprehensive and quantitative measurement of global lipid levels along with the estimation of circulating sugar, glycogen and protein content to gain insight in the context of weight change. We found that drastic alterations in the body weight of diseased flies is predominantly related to dysregulation of corresponding lipid level. Lipid level undergoes a rapid accumulation at an initial phase of disease i.e. highest at day 5 followed by severe drop at the end stage. Estimation of lipid levels from early to late stage of disease very well coincides with the excessive weight gain followed by progressive weight loss as evident from fresh weight measurements. Evaluation of carbohydrate and protein fraction further strengthened the impression that only lipid homeostasis correlates well with the weight modulation of diseased flies. In HD fly model, the carbohydrate moieties such as trehalose and glycogen were significantly higher at an earlier stage (day 3–5) but remain comparable to control flies at later stage. It is possible that the carbohydrate fractions might have contributed to the ongoing accumulation of lipids during the initial stage of disease that is otherwise not possible in normal condition. A substantial proportion of carbohydrates is converted into lipid molecules and stored in adipose tissue when body’s total carbohydrate stores increases considerably from their usual levels. The amount of lipid accumulated in these stores can far exceed that of glycogen and may elevate further to abnormal levels in case of any disturbed metabolic condition.

Usually, to maintain the cellular energy levels, circulating sugars are metabolized and exhausted first, followed by glycogen which can sustain the cellular energy needs for a comparatively longer period. Upon increased energy demands, the fat stores are broken down, as they can fulfill most of the cellular energy needs for maximum duration and only in case of extreme starvation, protein stores are metabolized. Present data also displays similar pattern of protein utilization upon disease progression. Diseased flies seem to bulk up the protein content during first week when carbohydrate and lipid moieties are adequately available; whereas upon pathologic decline of lipid levels towards the end stage, protein catabolism accelerates. Altogether, these findings lead us to the speculation that lipid metabolism remains predominantly altered leading to major weight changes in case of diseased flies.

Reports in HD mouse model demonstrate dysfunction in key metabolic tissues such as brown adipose tissue^29^ and white adipose tissue, with impairment in fat storage genes becoming more pronounced with disease progression^11^. *Drosophila* fat-body is functionally analogous to the vertebrate white adipose tissue. Intracellular organelles known as lipid droplets which serve as a storehouse of neutral lipids, mainly in the form of triacylglycerols (TAGs), enrich the adipose tissue. These lipid droplets serve as crucial intracellular biomarker to track abnormal accumulation or depletion of fat reserves. Therefore, we investigated the distribution of these lipid droplets in healthy and diseased condition that exhibited a marked difference in lipid droplet size with disease progression. However, above impairment in intracellular lipid accumulation strongly suggests an underlying dysregulation of metabolic integrity.

To further gain insight into the mechanistic basis of metabolic dysregulation seen in diseased flies, we investigated two groups of *Drosophila* neurons well known for their role in regulating lipid and carbohydrate metabolism. IPCs and APCs are present as discrete clusters of neurosecretory cells in brain and produce several insulin-like peptides and adipokinetic hormone, respectively. Several studies report that genetic ablation of these neuroendocrine cells induces metabolic abnormalities in the form of elevated carbohydrate levels^38^,^41^, hyperlipemia^41^ or reduced trehalose levels^40^. To discern, if the metabolic dysregulation we observed in HD flies is due to IPCs and APC neurons or a consequence of disease condition when mutant Htt is targeted in all the neurons, we targeted mutant Htt protein in these neurons. We evaluated the body weight, lipid levels and carbohydrate content of these flies and found significant alteration in the weight, lipid and carbohydrate reserves at specific ages. Interestingly, these alterations by targeting mutant Htt in IPCs and APC neurons only partially resembles the metabolic defects evident in HD flies when mutant Htt is targeted in all the neuronal population. Flies expressing mutant protein specifically in IPC cluster were able to maintain normal weight and lipid levels at most of the evaluated ages. Moreover, their glycogen and trehalose pattern further indicated presence of functional IPCs. On the contrary, mutant Htt expression in APCs leads to a significant decline in their fresh weight with age i.e. day 5 onwards, whereas the lipid stores underwent significant elevation at specific ages. Also, modulated levels of glycogen and disaccharide sugar trehalose in these flies altogether reflected altered APCs functioning. Mutant Htt expression in APCs resembled the metabolic alteration shown by mutant Htt expression in all the neurons in context of elevated lipid level, however, they did not display any gain in fresh weight. These results further strengthen the idea that metabolic defect evident in HD flies in the form of weight gain cannot be attributed singly to a particular neuronal subset, but, more likely results from dysfunction in multiple parameters such as muscle mass, fluid content, food intake or energy expenditure, when mutant Htt is targeted in all the neurons.

Our findings strongly suggest that mutant Htt expression exclusively in all the CNS and PNS neurons leads to fluctuation of body weight, carbohydrate and protein stores, global lipid levels along with the intracellular lipid deposits through its effect on the integrated process of metabolic homeostasis. Our results also support the idea that lipid metabolism remains centrally affected in HD, leading to altered body weight and subsequently a prevalent energy deficient background. These findings provide an insight in potentially controlling disease progression by checking metabolic dysregulation and thereby designing effective treatments to alleviate the suffering of affected individuals that ultimately might ensure better quality of life.

## Materials and Methods

### *Drosophila* Stocks and Crosses

Expression of transgene containing polyglutamine repeats is carried out by using the bipartite UAS-GAL4 expression system in transgenic *Drosophila*^31^. Fly lines used include transgenic stocks *w*; P{UAS-Httex1p Q20}, *w*; P{UAS*-*Httex1p Q93}4F1^32^, a pan-neuronal *w*; P{w^+mW^.^hs^=GawB}elavC155 driver, an adipokinetic hormone dAkh-GAL4 driver^42^ and an insulin-like peptide Ilp2-GAL4 driver^41^. Females from UAS-Httex1p Q93 line were crossed with males of *elav* Gal4, *Akh* Gal4 or *Ilp2* Gal4 line and the resulting female progeny (diseased) were used for all the assays. Cultures were reared at 25 °C and 65% humidity on corn agar media under a 12 h light: 12 h dark cycle. For control, females from *elav*>Httex1p Q20 or UAS-Httex1p Q93 not mated with specific drivers were used.

### Food Intake Assay

A colorimetric estimation of food intake was performed. For the food intake estimation, synchronized feeding third instar female larvae were sorted on the basis of gonad size. The male testis is transparent and much larger than female’s ovary, and is clearly visible in the opaque fat body. This clear space is not apparent in the female larvae. Group of 10 larvae from each condition were transferred into yeast paste with 4% Blue Dye #1 (Sigma Aldrich-861146) and left for feeding for 2 hours. After feeding, the larvae were thoroughly washed with ice cold water and homogenized in 200 μl of chilled 1X PBS. For adult food intake quantification, age matched flies from each condition were sorted into groups of 5 individuals and transferred to 2.5% blue dye supplemented food for continuous feeding at 25 °C. The flies were removed at an interval of 8 hours during morning, day and night time for dissecting their crop and midgut region of alimentary canal. The tissue was homogenized and the homogenate was centrifuged at 13,500 rpm for 10 minutes. 50 μl of supernatant was aliquoted in triplicates in 96 well plate and the absorbance was recorded at 625 nm using a spectrophotometer. Minimum three replicates per condition i.e., control and diseased were used for the assay.

### Glycogen Estimation

For glycogen estimation^46^, 4 flies/replicate were homogenized in 400 μl of 2% Na_2_SO_4_. 20 μl of the homogenate was aliquoted, mixed with 46 μl of 2% Na_2_SO_4_ and 934 μl of chloroform/methanol (1:1). The mixture was spun at 13,500 rpm for 10 minutes. Supernatant was discarded and pellet containing glycogen was air dried. 500 μl of anthrone reagent (0.2% anthrone in 72% sulphuric acid) (Sigma Aldrich-319899) was added and mixture was heated at 90 °C for 20 minutes and vortexed after every 5 minutes. The tubes were cooled on ice for 10 minutes and then returned to room temperature for 20 minutes. The absorbance was recorded at 620 nm. Carbohydrate concentration was calculated using a standard curve. Minimum three to five replicates per condition were used for the assay.

### Trehalose Estimation

Minimum 4 flies/replicate were homogenized in 500 μl of 70% ethanol and homogenate was centrifuged at 5,000 rpm at 4 °C to yield pellets. Dried pellets were re-suspended in 200 μl of 2% NaOH, heated at 100 °C for 10 minutes and cooled on ice^46^. 100 μl of the sample was then mixed with 750 μl anthrone reagent and anthrone reaction was performed as mentioned above in glycogen estimation. The absorbance of supernatant was recorded at 620 nm. Three to five replicates per condition were used for trehalose measurement.

### Protein Quantification

For protein estimation, 4 flies/replicate were homogenized in 400 μl of 2% Na_2_SO_4_ and 0.05% Tween 20 (1:1). 80 μl of the homogenate was aliquoted and 500 μl of 0.15% sodium deoxycholate was added. The mixture was kept on ice for 10 minutes and 1 ml of 3 M trichloroacetic acid was added. The tubes were spun at 8,500 rpm for 15 minutes at 4 °C. After discarding the supernatant, pellet was rinsed only once with 1 ml of 1M HCL, air dried and dissolved in 1.6 ml of Bicinchoninic acid (BCA) working reagent. The mixture was heated at 60 °C for 10 minutes and tubes were then kept on ice to stop further color development. The supernatant was aliquoted in multi-well plates and absorbance was recorded at 562 nm. Three to five replicates per condition were used for the protein estimation.

### Lipid Estimation

Freshly hatched, normal and diseased flies were etherized, sorted into groups of 10 flies each and transferred to pre-labeled clean empty dry vials. Samples were weighed in microgram using Citizen CM11 microbalance to obtain wet weight. Subsequently, the samples were dried in a preheated oven at 70 °C for 36 hours and weighed again to obtain dry weight. Ether soluble lipids were extracted by transferring intact dry flies to corresponding pre-labeled 1.5 ml microcentrifuge tubes containing 1 ml of diethyl ether at room temperature on a gel rocker. The lipids were extracted for 48 hours with three ether changes at an interval of 12 hours. After the last ether change, flies were dried for 2 hours at 30–35 °C and weighed to obtain lipid free weight of flies. The difference between dry weight and lipid free weight was considered as the total lipid content of the flies^47^,^48^. Similarly, water content was calculated by subtracting dry weight from fresh weight. Five replicate vials per condition were set up and lipid content was measured at 0, 3, 5, 7, 9, 11 and 13 days of age.

### Lipid Droplet Staining and Microscopy

For lipid droplet staining in adult fat body, abdomen of 5–6 flies of same age were dissected in ice-cold 1X PBS and fixed in 4% formaldehyde in PBT for 20 minutes at room temperature. After fixation, fat bodies from dorsal surface of abdomen were detached, fixed for an additional 10 minutes and rinsed thrice for 10 minutes each with 1X PBS. The tissue was incubated in freshly prepared 1:2000 dilution of 0.5 mg/ml Nile Red (Sigma Aldrich- N3013) with PBS for 30 minutes. Subsequently, tissue was rinsed twice with 1X PBS and mounted in Vectashield mounting medium with DAPI (Vector Labs) for nuclei staining. Samples were examined under Nikon Eclipse (Ni-E) fluorescence microscope and lipid droplets quantification was performed using NIS-Elements AR software. Minimum five samples per condition were analyzed.

### Statistical Analysis

For all the assays, mean value was used as the unit of analysis and the level of significance was determined using Student’s t-test. The graphical representations of all the results are indicative of mean value ± SEM.

## Additional Information

**How to cite this article**: Aditi, K. *et al*. Altered lipid metabolism in *Drosophila* model of Huntington’s disease. *Sci. Rep.* **6**, 31411; doi: 10.1038/srep31411 (2016).
